# Use of Batroxobin in Central and Peripheral Ischemic Vascular Diseases: A Systematic Review

**DOI:** 10.3389/fneur.2021.716778

**Published:** 2021-12-02

**Authors:** Duo Lan, Siying Song, Yunhuan Liu, Baolian Jiao, Ran Meng

**Affiliations:** ^1^Department of Neurology, Xuanwu Hospital, Capital Medical University, Beijing, China; ^2^Advanced Center of Stroke, Beijing Institute for Brain Disorders, Beijing, China; ^3^Department of China-America Institute of Neuroscience, Xuanwu Hospital, Capital Medical University, Beijing, China; ^4^Huadong Hospital, Fudan University, Shanghai, China

**Keywords:** batroxobin, vascular disease, ischemic, effects, mechanism

## Abstract

**Background and Purpose:** The mechanism of action of Batroxobin included the decomposition of the fibrinogen to fibrin degradation products (FDPs) and D-dimer and mobilization of endothelial cells to release endogenous nt-PA and to promote thrombolysis. This review aims to summarize current study findings about batroxobin on correcting cerebral arterial, venous, and peripheral vascular diseases, to explore the mechanism of batroxobin on anti-thrombosis process.

**Methods:** A thorough literature search was conducted utilizing the PubMed Central (PMC) and EMBASE databases to identify studies up to June 2021. Data from clinical studies and animal experiments about batroxobin were extracted, integrated and analyzed based on Cochrane handbook for systematic reviews of interventions approach and the Preferred Reporting Items for Systematic Review and Meta-Analysis Protocols (PRISMA-P), including the condition of subjects, the usage and dosage, research observation index and main findings.

**Results:** A total of 62 studies were enrolled in this systematic review, including 26 clinical studies and 36 animal experiments. The 26 clinical studies involved 873 patients with arterial ischemic events, 92 cases with cerebral venous thrombosis, 13 cases with cerebral cortical vein thrombosis, and 1,049 cases with peripheral vascular diseases. These patients included 452 males and 392 females aged 65.6 ± 5.53 years. The results revealed that batroxobin had broad effects, including improving clinical prognosis (*n* = 12), preventing thrombosis (*n* = 7), promoting thrombolysis (*n* = 6), and improving vascular cognitive dysfunction (*n* = 1). The effects of batroxobin on reducing neuronal apoptosis (*n* = 8),relieving cellular edema (*n* = 4), improving spatial memory (*n* = 3), and promoting thrombolysis (*n* = 13) were concluded in animal experiments. The predominant mechanisms explored in animal experiments involved promoting depolymerization of fibrinogen polymers (*n* = 6), regulating the expression of related molecules (*n* = 9); such as intercellular adhesion molecule, heat shock proteins, tumor necrosis factor), reducing oxidative stress (*n* = 5), and reducing inflammation response (*n* = 4).

**Conclusion:** Batroxobin can correct both arterial and venous ischemic diseases by promoting depolymerization of fibrinogen polymers, regulating the expression of related molecules, reducing oxidative stress, and reducing the inflammation response.

## Introduction

Batroxobin, isolated from *Bothrops atrox moojeni* venom, is widely used in clinical such as postoperative hemostasis of surgery because of its hemostatic effect (1–4). Batroxobin has also been investigated for the treatment of deep vein thrombosis and cerebral infarction as it promotes thrombolysis, prevents recurrence of thrombus, and provides neuroprotection (5–8). In recent years, the role of Batroxobin in cerebral venous thrombotic diseases has attracted more attention with two clinical articles proposing to study the clinical value of Batroxobin in cerebral venous thrombosis (CVT) and cerebral venous sinus thrombosis (CVST), respectively (9, 10). Batroxobin may promote venous sinus recanalization thrombosis recanalization, and is a potentially safe and effective adjunct therapeutic agent in patients with a high level of fibrinogen. Another small clinical study investigated the efficacy of Batroxobin in cerebral cortical vein thrombosis (CCVT). Batroxobin significantly improved the prognosis of patients with CCVT (11). All these studies prove that Batroxobin has a wide range of clinical applications. The mechanism of action of Batroxobin included the decomposition of the fibrinogen to fibrin degradation products (FDPs) and D-dimer (12, 13) and mobilization of endothelial cells to release endogenous nt-PA and to promote thrombolysis (14, 15). However, there is a lack of literature review that summarizes the clinical effects and related mechanisms of Batroxobin. Since there is a growing interest in studying Batroxobin as a treatment strategy in cerebral venous system diseases, our study aims to summarize the previous findings to provide a theoretical basis for the use of Batroxobin in cerebral venous system diseases and facilitate future research.

In this study, we review previous studies investigating Batroxobin in both clinical and experimental settings and summarize the most recent findings to provide a deep understanding of Batroxobin in treating thrombotic diseases. We also discuss the potential use of Batroxobin in the treatment of cerebral venous thrombotic diseases.

## Methods

### Search Strategy

A systematic review of the literature has been performed on PubMed Central (PMC) and EMBASE databases using the keywords “Batroxobin,” “animal study,” or “clinical study.” Our review includes studies published till June 2021 that investigated Batroxobin. Cochrane handbook for systematic reviews of interventions approach and the Preferred Reporting Items for Systematic Review and Meta-Analysis Protocols (PRISMA-P) was followed accordingly (Supplementary Table 1).

### Study Selection

**Figure 1:**
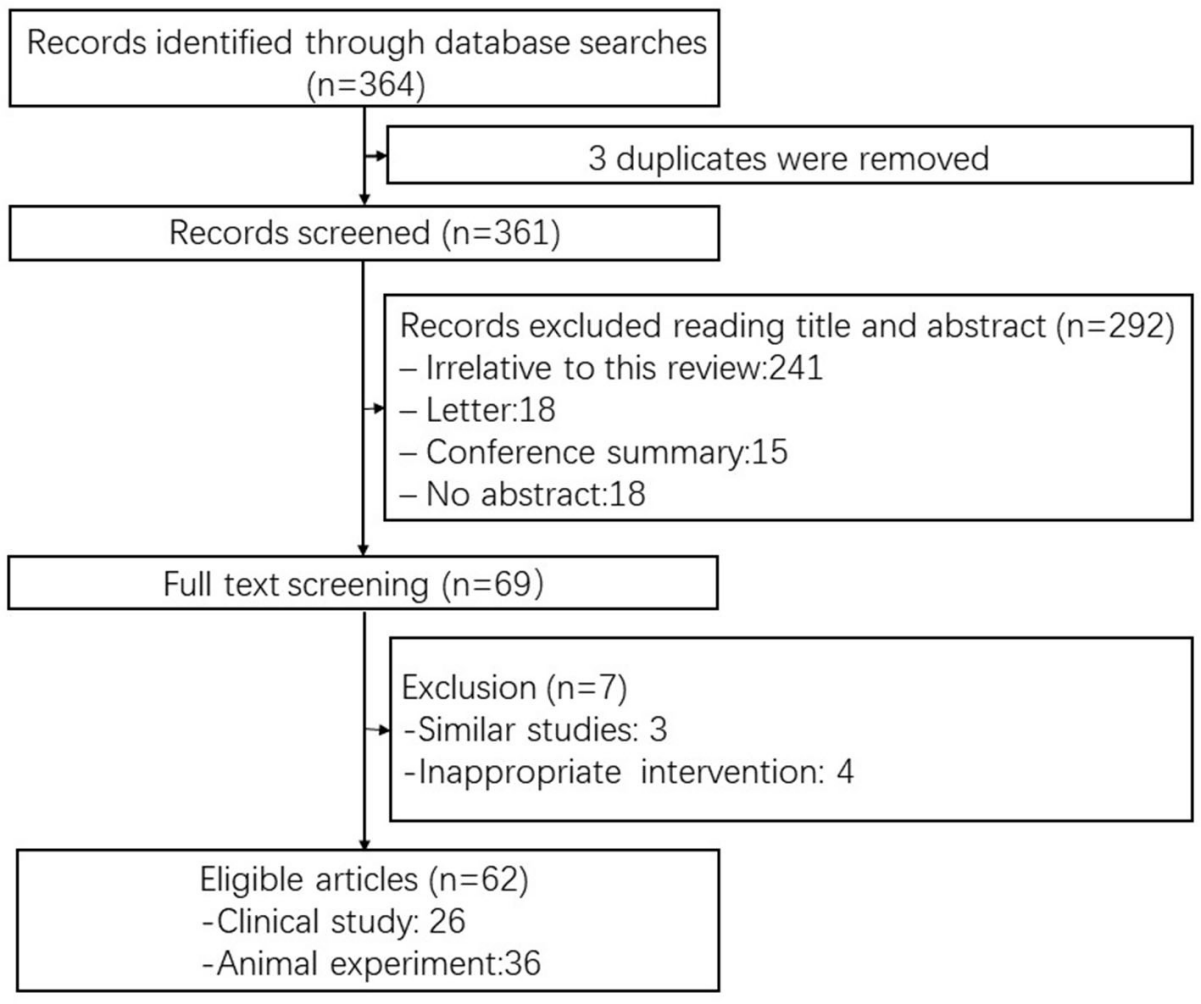
Flow diagram of the study selection process.

Clinical (prospective and retrospective) and experimental studies that evaluated the efficacy of Batroxobin were included. Studies not related to vascular system diseases and their complications were excluded. Conference abstracts, reviews, case reports, and letters were also not included in the analysis. If two or more studies had duplicate or overlapping data, then the study with the larger sample size and more detailed data was selected. Two reviewers (D-L and SY-S) independently performed the study selection and any disagreements were resolved by discussion (Figure 1).

### Data Extraction

Two authors (D-L and SY-S) extracted data from the selected studies, which was evaluated by another author (BL-J). The data were further extracted and summarized as follows: the name of the first author, year of publication, country, study characteristics (sample size and research type), subject characteristics (population and animal status, comorbid status and animal model type), detailed information of Batroxobin use, primary outcome and other main findings. All disagreements were resolved by consensus.

### Outcomes

The main outcomes of the clinical trials in this review were coagulation indicators, improvement of neurological function, and thrombus recanalization and recurrence. The main outcomes of animal experiments were histopathological indexes and blood factor indexes.

## Results

Sixty-two studies, including 26 clinical studies and 36 animal experiments, were selected for the systematic review. The specific screening process is shown in Figure 1 and detailed information about the selected studies is listed in Tables 1, 2.

**Table 1:** Application of Batroxobin in clinical studies.

**References**	**Country**	**Population**	**Study type**	Sample size, age^*^	**Gender** **(F/M)**	**Intervention and dosage**	**Outcome evaluation**	**Main findings^**#**^**
Song et al. (11)	China	CCVT	Case-control	C: 9 (30.4 ± 14.5) I: 4 (32.8 ± 4.0)	8/5	C: LMWH bridged with Warfarin I: LMWH bridged with Warfarin + Batroxobin Dosage: 10 BU followed by 5 BU every other day, iv. drip.	•PGIC •Time to symptom relief •Recanalization rate •Recurrence rate	•Improvement on PGIC scores •Decreased time to symptom relief •Accelerated recanalization •Decreased recurrent rate of CCVT
Ding et al. (10)	China	CVT	Case-control	C: 10 (39.2 ± 21.5) I: 21 (29.8 ± 14.5)	16/15	C: LMWH bridged with Warfarin I: LMWH bridged with Warfarin + Batroxobin Dosage:10 BU followed by 5 BU every other day, iv. drip.	•Recanalization degree •NIHSS •mRS •Adverse event	•Increased recanalization rate •Increased the rate of stenosis reversion •No statistical difference on NIHSS and mRS
Ding et al. (9)	China	CVST	Case-control	C: 38 (36.3 ± 15.3) I: 23 (29.8 ± 14.5)	30/31	C: LMWH I: LMWH + Batroxobin Dosage:10 BU followed by 5 BU every other day, iv. drip.	•mRS •NIHSS •Recanalization rate •Hemorrhage rate •TT •DD •Fg	•Increased recanalization rate •Improvement on NIHSS •No increased risk of intracranial hemorrhage •Prolongation of TT, increased DD, and decreased Fg
He et al. (16)	China	AIS	Case-control	C: 47 (55.72 ± 9.84) I: 43 (58.91 ± 11.64)	NA	C: Aspirin + Atorvastatin + Batroxobin I: Aspirin + Atorvastatin +Batroxobin + TCD Dosage: 10 BU, iv. drip.	•Hemodynamic monitor •NIHSS •TIBI •BI •Recurrence rate	•Improvement on NIHSS and BI •Reduction of stroke recurrence rate
Wu et al. (17)	China	AIS	Case-control	C: 43 I: 43	NA	C: Batroxobin I: Batroxobin + Edaravone	•NDS	•Lower NDS •Higher general effective rate
Ren et al. (18)	China	AIS	Case-control	50	NA	C: Batroxobin I: Batroxobin + Edaravone Dosage: 10 BU followed by 5 BU every other day for 4 times, iv. drip.	•ESS •BI •Fg	•Improvement of ESS •Decreased Fg •Higher effective rate
Hao et al. (19)	China	AIS	Case-control	45	NA	C: Batroxobin +Normal temperature I: Batroxobin +Local mild hypothermia	•ESS •Effective rate	•Improvement of ESS •Higher effective rate
Wang et al. (20)	China	AIS	Case-control	80	NA	C: Batroxobin I: Batroxobin + Edaravone Dosage: 10 BU followed by 5 BU every other day for 3 times, iv. drip.	•NDS •Fg	•Decreased Fg in both group •Higher effective rate
Xu et al. (7)	China	AIS/TIA with hyperfibrinogenemia	Case-control	C: 60 (65 ± 7.3) I: 52 (66.1 ± 8)	85/27	C: Saline I: Batroxobin	•Recurrence rate	•Reduction of stroke recurrence rate
Gusev et al. (21)	Russia	AIS	Case-control	C: 45 I: 45	NA	C: Standard therapy I: Standard therapy+ Batroxobin Dosage: 10 BU followed by 5 BU every other day for 3 times, iv. drip.	•Physical examination •Fg •DD	•Improvement on symptoms of motor disability. •Decreased Fg •Increased DD
Yu et al. (22)	China	AIS	Case-control	C: 108 I: 106	NA	C: Conventional therapy I: Conventional therapy+ Batroxobin	•Effective rate	•Quicker function recovery •Shorter course of the disease
Tanahashi et al. (23)	Japan	AIS	Retro	C:8 I:8	NA	C: Batroxobin I: Batroxobin Dosage: C: 5 BU for one time, iv. drip. I: 10 BU for one time, iv. drip	•Fg	•Decreased RBC-A •Decreased Fg
Zhai et al. (24)	China	VCI	Case-control	C: 40 I: 40	NA	C: Aspirin I: Aspirin + Batroxobin Dosage: 5 BU for 4 times a week, iv. drip.	•MMSE •ADL	•Improvement on MMSE and ADL
Chen et al. (25)	China	DVT after PCLR	Case-control	128	36/92	LMWH + Batroxobin	•Recanalization rate •DD	•Increase in DD •Increase in recanalization rate •No PE and hemorrhage
Ye et al. (26)	China	DVT after ACLR	Retro	195	48/123	Batroxobin Dosage: 5 BU for 3 times for distal DVT and 3 to 5 times for proximal DVT, iv. drip.	•Recanalization rate	•Increase in recanalization rate •No PE and hemorrhage
Qin et al. (6)	China	DVT in AIS	Case-control	C:47 (74 ± 6) I:10 (75 ± 8)	33/24	Batroxobin Dosage: 10 BU followed by 5 BU for 3–14 days according to the DVT symptom, iv. drip.	•Recanalization rate	•Increase in recanalization rate •No PE and hemorrhage
Zhang et al. (5)	China	DVT	Retro	15	NA	Batroxobin + LMWH + Aspirin Dosage: 10 BU followed by 5 BU for 14 days, iv. drip.	•Recanalization rate •Fg	•Reduction of Fg level •Improvement of symptoms •Increased recanalization rate •Increased CD34þ/CD31þ cells
Wang et al. (27)	China	DVT	Retro	I A1: 25(48 ± 16) I A2: 23(49 ± 15) I B: 14(52 ± 15) I C1: 25(50 ± 15) I C2: 25(48 ± 15) I D: 15(46 ± 15)	66/61	I A1: Batroxobin I A2: Batroxobin I B: LMWH I C1: Batroxobin + LMWH I C2: Batroxobin + LMWH I D: Urokinase +LMWH Dosage: I A1:10 BU (1-day) followed by 5 BU, iv. drip I A2: 10 BU (1-day) followed by 5 BU, micro pump I C1:10 BU (1-day) followed by 5 BU, iv. drip I C2: 10 BU (1-day) followed by 5 BU, micro pump	•Fg •Complication •The perimeter of the thigh and calf	•The combination usage of Batroxobin + LMWH achieved the best efficacy •The safety of Batroxobin given in micro pump was much better.
Xue et al. (28)	China	Arterial angioplasty	Case-control	C: 26 I: 26	NA	C: Aspirin I: Aspirin + Batroxobin Dosage:5 BU every other day for 6 times, iv. drip.	•ABI •Restenosis rate	•Decreased restenosis rate •Increased ABI
Wang et al. (29)	China	Arterial angioplasty	Case-control	C:26 (70.92 ± 6.53) I: 26 (69.62 ± 7.75)	24/22	C: Aspirin I: Aspirin + Batroxobin Dosage:5 BU every other day for 6 times, iv. drip.	•Restenosis rate •Clinical symptom •Relief rate •ABI	•Decreased restenosis rate •Increased ABI
Yasunga et al. (30)	Japan	PAT	Retro	8	NA	Batroxobin Dosage: 0.4–0.8 BU/kg, iv. drip.	•Fg •PT •APTT	•Decreased blood viscosity, Fg, and plasminogen •Prolongation of PT, APTT, and plasma clot lysis time •Reduction of factor II, factor VII and α2 macroglobulin
Li et al. (31)	China	Arterial angioplasty	Case-control	C: 55 (70.60 ± 7.10) I: 46 (69.54 ± 6.91)	56/45	C: Aspirin I: Aspirin + Batroxobin Dosage:5 BU every other day for 6 times, iv. drip.	•Restenosis rate	•Decreased restenosis rate •Increased limb salvage-survival rates
Wang et al. (32)	China	Arterial angioplasty	Case-control	C: 60 (70.7 ± 7.40) I: 51 (69.49 ± 6.93)	64/47	C: Aspirin I: Aspirin + Batroxobin Dosage:5 BU every other day for 6 times, iv. drip.	•Restenosis rate •Limb salvage and survival rates •ABI	•Decreased restenosis rate •Increased limb salvage-survival rates •Increased ABI
Xiao et al. (33)	China	ACS after stenting	Case-control	C: 20 I: 20	NA	C: Aspirin + Clopidogrel I: Aspirin + Clopidogrel+ Batroxobin Dosage: 10 BU for one time, iv. drip.	•CRP	•Decreased CRP •Decreased restenosis rate
Sakamoto et al. (34)	Japan	AF	Self -control	Group 1: 15 (66 ± 9) Group 2: 13 (68 ± 7) Group 3: 8 (74 ± 11)	9/27	Groups divided by grades of atrial spontaneous echo contrast Group 1: mild Group 2: moderate Group 3: severe Dosage: 0.2 BU/kg, iv. drip.	•Fg •Whole blood viscosity	•Improvement on blood rheology •Decreased blood cell aggregation •Prevention of atrial thrombus formation
Choi et al. (35)	Japan	Healthy subjects	Case-control	C: 6 (28.5 ± 7.4) I1: 6 (26.3 ± 7.5) I2: 6 (29.3 ± 6.2) I3: 6 (27.2 ± 2.9)	NA	C: Placebo I1: Batroxobin I2: Batroxobin I3: Batroxobin Dosage: I1: 2.5 BU, iv. drip. I2: 5 BU, iv. drip. I3: 10 BU, iv. drip.	•PT •APTT •TT •Safety	•No significant changes in PT or APTT occurred •A dose range of 2.5–10.0 BU/2.0 mL was well tolerated •Decreased Fg •Prolongation of TT.

* *Mean ± standard deviation*.

# *Compared with control group*.

*CCVT, Cerebral cortical vein thrombosis; CVST, Cerebral venous sinus thrombosis; CVT, Cerebral venous thrombosis; DVT, Deep venous thrombosis; PCLR, Posterior cruciate ligament reconstruction; ACLR, Anterior cruciate ligament reconstruction; AIS, Acute ischemic stroke; TIA, Transient ischemic attack; VCI, Vascular cognitive impairment; ACS, Acute coronary; CABG, Coronary artery bypass graft; PLIF, Posterior lumbar interbody fusion; PAT, Peripheral arterial thrombosis; AF, Atrial Fibrillation. Retro, Retrospective; C, Control; I, Intervention; LMWH, Low molecular weight heparin; BU, Batroxobin unit; Iv. drip, intravenous drip; PGIC, Patient global impression of change; mRS, Modified Rankin Scale; NIHSS, National Institute of Health Stroke Scale; DD, D-dimer; US, Ultrasound; PE, Pulmonary embolism; RBC-A, Erythrocyte aggregability; TCD, Transcranial Doppler; TIBI, Thrombolysis in brain ischemic score; BI, Barthel index; NDS, Neurological deficit scale; ESS, European stroke scale; MMSE, Mini-mental state examination; ADL Activities of daily living; CRP, C-reactive protein; NS, Normal sodium; DM, Diabetes mellitus; ABI, Ankle-brachial index; PT, Prothrombin time; APTT, Activated partial thromboplastin time; Fg, Fibrinogen; FDP, Fibrinogen degradation product*.

**Table 2:** Application of batroxobin in animal experiments.

**References**	**Country**	**Animal model**	**Sample size**	**Intervention and dosage**	**Outcome evaluation**	**Main findings^**#**^**
Li et al. (36)	China	ACI (Gerbil)	NA	Sham operation group Control group I1: Batroxobin I2: Batroxobin + Edaravone	•Histological assessment	•Reduction of apoptosis of neurons
Hu et al. (37)	China	ACI (Rat)	120	C: No intervention I1: Batroxobin I2: Urokinase I3: Batroxobin + Urokinase Dosage: 5 BU/kg	•Intracranial bleeding •Histological assessment Neurological function	•Reduction of the cerebral infarct volume ratio. •No increased risk of intracranial hemorrhage
Wu et al. (38)	China	ACI (Rat)	NA	C: No intervention I: Batroxobin	•GAP-43 •Histological assessment	•Promotion of the expression of GAP-43 in infarction •Amelioration of the pathological changes in infarction
Wu et al. (39)	China	ACI (Rat)	NA	C: No intervention I: Batroxobin	•Cognitive function •NCAM	•Improvement on spatial memory disorder •Regulate the expression of NCAM
Wu et al. (40)	China	ACI(Rat)	NA	C: No intervention I: Batroxobin	•Cognitive function •HSP32 •HSP70	•Improvement on spatial memory disorder •Down-regulated HSP32 and HSP70
Wu et al. (40)	China	ACI (Rat)	NA	C: No intervention I: Batroxobin	•Cognitive function •C-Jun	•Improvement on spatial memory disorder •Down-regulated expression of c-Jun
Qun et al. (41)	China	ACI (Gerbil)	NA	C: No intervention I: Batroxobin Dosage: 8 BU/kg	•Histological examination •Oxidative stress product	•Ameliorated neurologic deficits •Increased surviving numbers of pyramidal cell •Reduction of hydroxyl radical production
Namikata et al. (42)	Japan	ACI (Rat)	NA	C: No intervention I: Batroxobin	•Histological examination •Neurological function	•Reduction of the degree of the edema and the size of infarction. •Relieved symptoms neurological deficits •Decreased mortality
Xu et al. (43)	China	Cerebral IR (Gerbil)	45	Groups divided by drug use frequency I1: Three times I2: Five times I3: Seven times Dosage: 8 BU/kg	•Histological examination	•Reduction of the number of apoptotic neurons •A dose-dependent neuroprotective effect
Kang et al. (14)	China	Cerebral IR (Rat)	32	C: No intervention I: Batroxobin Dosage: 0.3 BU/kg	•TNF-α •Histological examination	•Inhibition of the excessive increase of TNF-α. •Relieved cellular edema •Reduced pyknosis of nerve cells •No micro-thrombosis
Zhang et al. (44)	China	Cerebral IR (Gerbil)	60	Sham-operated group Ischemia control group Normothermia group Hypothermia group Batroxobin group Hypothermia + Batroxobin group Dosage: 8 BU/kg	•Oxidative stress product	•Increased SOD activities •Reduction of the MDA content
Wu et al. (45)	China	Cerebral IR (Rat)	36	C: Saline I: Batroxobin	•Histological examination	•Decreased apoptotic cells •Relieved the neuronal damage
Chen et al. (8)	China	Cerebral IR(Gerbil)	32	C: No intervention I: Batroxobin with different dose	•ATP levels •Neuron survival •Behavioral tests	•Decreased the neuron death •Increased ATP levels in the infarcted area •Decreased 2,3-DHBA
Yi et al. (46)	China	Cerebral IR (Rat)	NA	C: No intervention I: Batroxobin Dosage: 8 BU/kg	•Purine metabolites	•Decreased adenosine, inosine, hypoxanthine, and xanthine in ECF
Qi et al. (47)	China	ICH(Rat)	NA	Groups divided by dosage I1: Batroxobin 4 BU/kg group I2: Batroxobin 8 BU/kg group I2: Batroxobin 16 BU/kg group	•Histological examination •Oxidative stress product	•Improvement of neuroethology scale of the rats •Relieved histiocyte edema and bleeding. •Decreased water content, MDA, and free Ca2+ concentration •Increased SOD activities
Wu et al. (15)	China	ICH (Rat)	NA	C: No intervention I: Batroxobin	•Histological assessment •Immune factor	•Attenuated brain edema formation in ICH rats. •Down-regulated ICAM-1 in the perihematomal area •Down-regulated complement C3d and C9 expression in the perihematomal area.
Li et al. (48)	China	Nigrostriatal pathway injury (Rat)	24	C: Saline I: Batroxobin	•Histological examination •Neurological function •Immunohistochemistry	•Improvement on motor function •Reduction of neuronal apoptosis and inflammation at the acute stage •Attenuated scar formation and lesion size
Liu et al. (49)	China	Anoxic damage (Rat)	NA	C: No intervention I: Batroxobin	•Histological assessment •HSP70	•Neuroprotective effect on anoxic damage of hippocampal neurons. •Down-regulated HSP70
Inoue et al. (50)	USA	Demyelinating disease (Rat)	52	C: Saline I: Batroxobin Dosage: 30 BU/kg	•Clinical sign •Fg deposition •Coagulation test	•Delayed the onset •Decreased the severity of the demyelinating disease •Decreased the mean clinical severity of the disease
Yang et al. (51)	China	EAE (Rat)	36	Batroxobin Dosage: 30 BU/kg	•Histological examination •Coagulation test	•Ameliorated the clinical manifestation •Delayed the course •Reduction of inflammation and demyelination •Decreased deposition of Fg •No effect on plasma Fg •Down-regulated the expression of p-Akt •Up-regulated the expression of MBP
Yu et al. (52)	China	SCI (Rat)	90	C: No treatment I: Batroxobin	•BBB scores •Histological assessment •VEGF	•Increased expression of VEGF •Reduction of the number of apoptotic cells •Improvement the BBB scores
Fan et al. (47)	China	SCI (Rat)	NA	Groups divided by dosage C: Saline I1: Batroxobin 2 BU/kg I2: Batroxobin 4 BU/kg	•Coagulation test •Neurological function •Histological examination	•Decreased Fg •In I1 group (2 BU/kg), •Increased blood flow •Increased survival rate of neurons •Reduced lesion size •Alleviation of astrocyte and activation of microglial cell •Increased functional recovery
Jiang et al. (53)	China	AMI (Dog)	47	C: No intervention I: Batroxobin	•CK •LDH •Oxidative stress product •Histological assessment	•Decreased mortality •Decreased MDA and CK/LDH •Improvement on myocardial function
Gao et al. (54)	China	AMI (Dog)	NA	C: No intervention I: Batroxobin Dosage: 2 BU/kg	•CBF •Small coronary resistance	•Dose-dependent increase in CBF •Decreased small coronary resistance
Tomaru et al. (55)	Japan	AMI(Dog)	111	I1: Batroxobin I2: Aspirin I3: Heparin Dosage: 2 BU/kg	•Restenosis rate	•In I1 group (2BU/kg), •Complete prevention of restenosis.
Seon et al. (56)	Korea	Femoral artery hemorrhage (Rat)	120	Groups divided by dosage C: r-Batroxobin 0 BU/25 cm^2^ I1: r-Batroxobin 10 BU/25 cm^2^ I2: r-Batroxobin 25 BU/25 cm^2^	•Hemostatic activity •Coagulation test	•Facilitated erythrocyte aggregation and Fg clot formation •Accelerated blood coagulation
Seon et al. (56)	Korea	Femoral artery hemorrhage (Rat)	NA	C: Collagen I: Collagen + Batroxobin	•Hemostatic activity	•More rapidly controlled excessive bleeding with r-Batroxobin •Improved the effect of other hemostatic dressing.
You et al. (57)	Korea	Liver injury (Rat)	NA	Groups divided by dosage C: Batroxobin 0 BU/ml I1: Batroxobin 5 BU/ml I2: Batroxobin 10 BU/ml	•Coagulation test	•Facilitated blood coagulation. •Dose-dependent response of hemostasis
Tomaru et al. (58)	Japan	Hind limb artery injury (Dog)	67	I1: Heparin I2: Argatroban I3: Batroxobin Dosage: 0.05 BU/kg	•The rate of a thrombotic event •Coagulation test	•In I3 group •Safer and more effective in preventing thrombosis •No change of APTT and Fg
Masuda et al. (59)	Japan	Hind limb ischemic injury (rat)	NA	C: Saline I: Batroxobin	•Histological assessment •Blood perfusion	•Inhibition of NETs with Fg deposition and subsequent tissue damage •Acceleration of tissue repair •Expedited vascular regeneration •Acceleration of skeletal muscle regeneration
Tomaru et al. (54)	Japan	PAT (dogs)	73	C: Saline I1: Heparin I2: Argatroban I3: Batroxobin Dosage: 0.05 BU/kg	•Coagulation test •The reduction of •thrombotic stenosis	•In I3 group •Reduction of plasma Fg •Prevention of thrombosis
Tomaru et al. (55)	Japan	PAT(Rat)	23	C: No intervention I: nt-PA I2: nt-PA + Heparin I3: nt-PA + Batroxobin	•The rate of •recanalization	•Enhancement of thrombolytic effect of nt-PA.
Yoshikawa et al. (60)	Japan	DIC (Rat)	110	C: Saline I: Batroxobin Dosage: 200 BU/kg	•Fg •PT •APTT	•Reduction of plasma Fg •Increase in Fg degradation products •Prolongation of PT and APTT •Reduction of Blood cell counts, platelet counts, and hematocrit level
Markwardt et al. (61)	German	DIC (Rat)	NA	C: Saline I: Batroxobin	•Fg •Platelet counts •Hemoglobin	•Reduction of plasma Fg •Reduction of platelet counts •Increase in hemoglobin
Huang et al. (62)	China	Atherosclerosis (Rabbit)	50	C: Saline I: Batroxobin	•Stability evaluation vascular plaque	•Stabilization of atherosclerotic plaque
Wang et al. (63)	China	Healthy rat	40	Groups divided by dosage I1: Batroxobin 3 BU/ml I2: Batroxobin 10 BU/ml I3: Batroxobin 30 BU/ml	•SMC migration	•Inhibition of human vascular SMC migration

# *Compared with control group*.

*AMI, Acute myocardial ischemia; ACI, Acute cerebral ischemia; PAT, Peripheral arterial thrombosis; I/R, ischemia/reperfusion; DIC, Disseminated intravascular coagulation; SCI, Spinal cord injury; ICH, Intracerebral hemorrhage; r-batroxobin, Recombinant batroxobin; nt-PA, Native tissue type plasminogen activator; GAP-43, Growth-associated protein-43; CK, Creatine kinase; LDH, Lactate dehydrogenase; MDA, Malondiadehyde; CBF, Coronary blood flow; RL, Large coronary resistance; Fg, Fibrinogen; ATP, Adenosine triphosphate; ICAM-1, Intercellular Adhesion Molecule 1; TNF-α, Tumor necrosis factor alpha; ECF, Extracellular Fluid; VEGF, Vascular endothelial growth factor; NCAM, Neural cell adhesion molecule; HSP, Heat shock proteins; MBP, Maltose-binding protein; p-Akt, Phospho-Akt (Ser473); SOD, Superoxide dismutase; SMC, Smooth muscle cell; Fg, Fibrinogen; PT, Prothrombin time; APTT, Activated partial thromboplastin time; BBB, Basso-Bettie-Bresnahan; NETs, Neutrophil extracellular traps*.

### Clinical Studies

Two clinical studies, including 31 and 61 subjects, evaluated the efficacy of the combination of Batroxobin and anticoagulation in cerebral venous thrombosis (CVT) and cerebral venous sinus thrombosis (CVST), respectively (9, 10). Higher recanalization rates were found in both Batroxobin groups (adjusted OR [95% CI] of 2.5 [1.1–5.0]; adjusted OR [95%CI] of 8.10 [1.61–40.7], respectively) compared with the control groups, especially in patients with high levels of fibrinogen (adjusted OR [95% CI] of 4.7 [1.4–16.7]). The results of the two studies were inconsistent in concluding whether Batroxobin improved neurological deficits. National Institute of Health Stroke Scale (NIHSS) scores significantly improved at discharge in the Batroxobin group [0(0, 4.25)−5(2, 11), *p* = 0.036] compared with the baseline in only one study (9). A clinical study with 13 patients evaluated the effectiveness of Batroxobin in acute cerebral cortical vein thrombosis (CCVT) (11). Compared with the non-Batroxobin group, the Batroxobin group achieved a significantly improved prognosis, evaluated by the global impression of change (PGIC) (*p* = 0.030) in patients.

Ten studies investigated the efficacy of Batroxobin in patients with acute ischemic stroke (AIS). Six studies reported significant improvement of nerve function evaluated by NIHSS (*n* = 1), Neurological deficit scale (NDS) (*n* = 2), European stroke scale (ESS) (*n* = 2) (16–19, 21, 22). Two studies reported a positive association between Batroxobin and prevention of recurrence of stroke (7, 16). Three studies concluded that Batroxobin significantly decreases the level of fibrinogen and increases the level of D-dimer (18, 20, 23).

One study investigated the effect of Batroxobin in improving vascular cognitive dysfunction (24). Significant differences were observed in Mini-mental state examination (MMSE) and activities of daily living (ADL) scores compared with baseline.

The application of Batroxobin was also tested in peripheral vascular disease, deep venous thrombosis (DVT) (*n* = 5) (5, 25–27, 64), peripheral arterial thrombosis (PAT) (*n* = 5) (4, 28–30, 32), trial fibrillation (AF) (*n* = 1) (34) and healthy subjects (*n* = 1) (35). In all five DVT studies, Batroxobin promoted the recanalization of thrombosis and decreased the occurrence of restenosis of PAT. Batroxobin promoted favorable clinical outcomes in patients with peripheral arteriovenous thrombosis, evaluated by ankle-brachial index (ABI). Coagulation tests with Batroxobin showed a significant decrease in FIB (5, 27, 30) and prolongation of thrombin time (TT) (35) in these studies. Batroxobin also affected other clotting indicators such as prothrombin time (PT) and activated partial thromboplastin time (APTT), but the exact role is controversial (30, 35).

### Animal Experiments

In animal experiments, the main objective was to understand the central vascular damage model that is involved in acute cerebral ischemia (ACI) [*n* = 8; rat(*n* = 6) and gerbil (*n* = 2)], cerebral ischemia-reperfusion (IR) [*n* = 6; rat(*n* = 3) and gerbil(*n* = 3)], intracerebral hemorrhage (ICH) (*n* = 2; rat), and spinal cord injury (SCI) (*n* = 2; rat). Four studies also assessed the effect of Batroxobin in the rat models of anoxic damage, nigrostriatal pathway injury, demyelinating disease, and experimental autoimmune encephalomyelitis. Twelve studies showed that Batroxobin reduces neuronal apoptosis (*n* = 8) (8, 36–39, 41, 43, 48) and relieves cellular edema (*n* = 4) (14, 15, 42, 65) by promoting the expression of growth-associated protein-43 (GAP-43) (38), increasing the level of adenosine triphosphate (ATP) (8), decreasing the hydroxyl radical production (41, 44, 65), down-regulating the heat shock proteins (HSP) (49), and down-regulating complement expression (15). Three experiments concluded that Batroxobin significantly improved the spatial memory and cognitive function in rats by regulating the expression of HSP32, HSP70 and neural cell adhesion molecule (NCAM) (40, 45, 66).

The peripheral vascular model included three bleeding models; the rest were all ischemic models including acute myocardial ischemia (AMI) (*n* = 3; dog), disseminated intravascular coagulation (DIC) (*n* = 2; rat), peripheral artery thrombosis/ischemic injury [*n* = 4; dog(*n* = 2) and rat (*n* = 2)], and atherosclerosis (*n* = 1; rabbit). Four experiments confirmed that Batroxobin decreased fibrinogen levels (47, 54, 60, 61). Further, Batroxobin decreased blood counts, platelet counts, and hematocrit level (60, 61). Two experiments showed that Batroxobin also promoted coagulation (57, 67). Other reports showed that Batroxobin also participated in stabilizing the atherosclerotic plaque, inhibiting human vascular smooth muscle cell migration, accelerating tissue repair, and expediting vascular regeneration (59, 62, 63).

## Discussion

**Figure 2:**
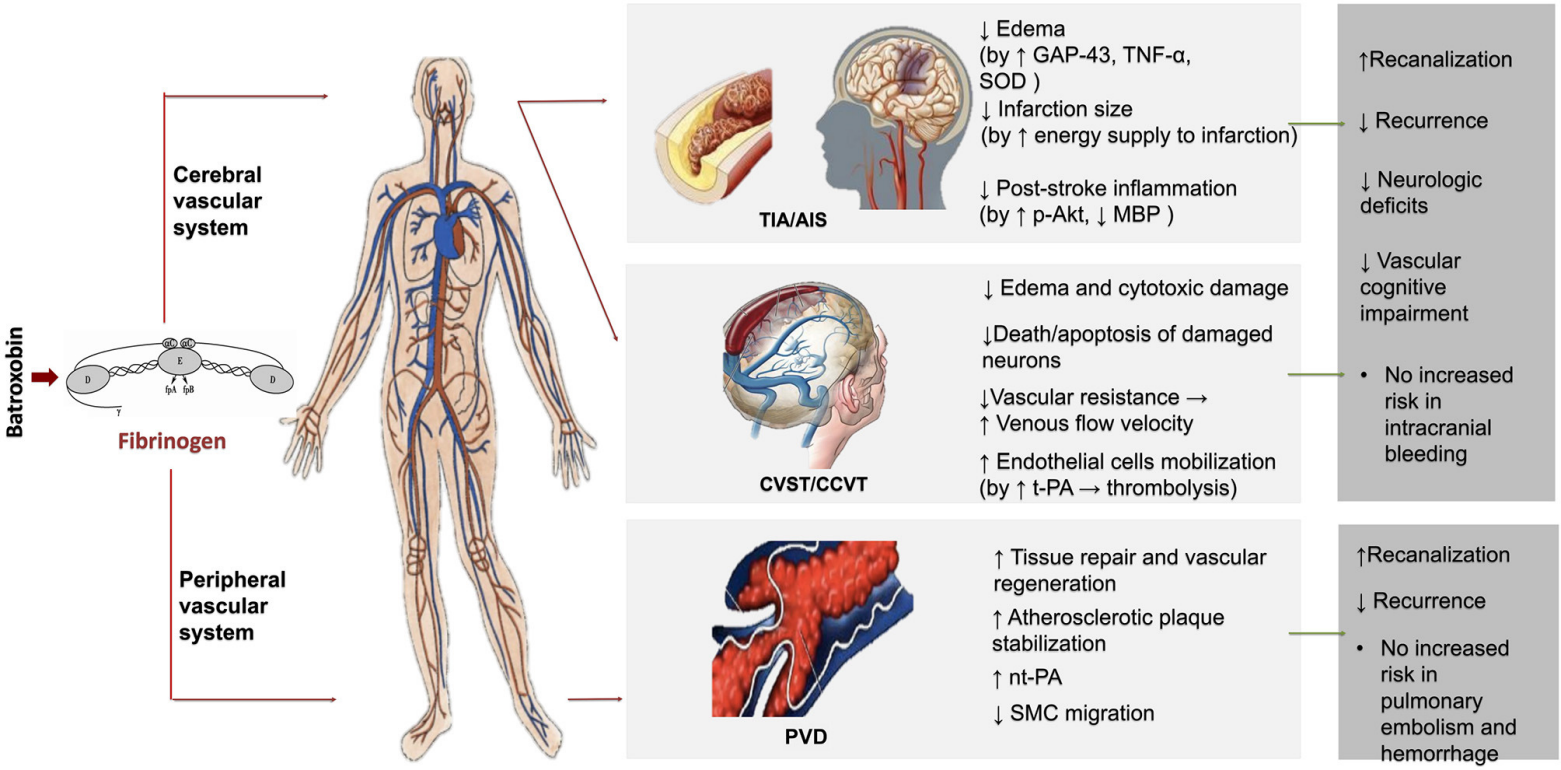
The possible mechanisms and clinical application of batroxobin. TIA, Transient ischemic attack; AIS, Acute ischemic stroke; CVST, Cerebral venous sinus thrombosis; CCVT, Cerebral cortical vein thrombosis; PVD, Peripheral vascular disease; GAP-43, Growth-associated protein-43; TNF-α, Tumor necrosis factor alpha; SOD, Superoxide dismutase; nt-PA, Native tissue type plasminogen activator; MBP, Maltose-binding protein; p-Akt, Phospho-Akt (Ser473); SMC, Smooth muscle cell.

Our review for the first time summarizes the clinical applications and possible mechanisms of Batroxobin by systemically reviewing current clinical and experimental studies (Figure 2).

### The Application of Batroxobin in Central Vascular Disease

The effectiveness of Batroxobin in promoting recanalization (9, 10) and preventing recurrence (7, 16) of thrombus in all patients with ischemic disease, including cerebral venous sinus thrombosis (CVST) or acute ischemic stroke (AIS), were supported by several studies. In addition to its benefit for recanalization and secondary stroke prevention, treatment with Batroxobin also improved the neurologic deficits which secondary to CVST or AIS (1, 8, 14–16, 23, 67). A case-control study showed that Batroxobin in combination with aspirin improved vascular cognitive impairment (VCI) (24). Batroxobin did not increase the relative risk of any adverse events, including intracranial bleeding (9), compared with the control group.

In animal models of cerebral ischemia or ischemia-reperfusion, Batroxobin reduced the number of apoptotic neurons (8, 14, 36, 39, 41, 43), the degree of edema (14, 42) and the size of infarction (37–39, 42, 46) and the occurrence of micro-thrombosis (14). Batroxobin may produce these effects through a variety of pathophysiological mechanisms, including promotion of the expression of growth-associated protein-43 (GAP-43) (38), inhibition of the excessive increase of Tumor necrosis factor-alpha (TNF-α) (14), increase of the Superoxide dismutase (SOD) activities (44), reduction of oxygen-free damage (41, 44) and increase of the energy supply to the infarct area (8). Batroxobin increases the expression of neural cell adhesion molecule (NCAM) and downregulates the generations o9f heat shock proteins (HSP), such as HSP32 and HSP70, and c-jun, thereby, improving spatial memory disorder (40, 45, 66). In the models of intracerebral hemorrhage (ICH), Batroxobin effectively attenuated brain edema formation and decreased bleeding, possibly by decreasing the concentration of malondialdehyde (MDA) and free Ca2+, increasing the SOD activities and down-regulating the expression of Intercellular Adhesion Molecule 1 (ICAM-1) and complements, such as C3d and C9 (15, 65). Batroxobin was also effective in other animal models of central disease, including nigrostriatal pathway injury, widespread anoxic damage, demyelinating disease and spinal cord injury (SCI) (47–52). Batroxobin also attenuates the scar formation (48), display a direct neuroprotective effect on anoxic neuron (49) and delay the onset and the course of demyelinating disease; (50, 51) possible mechanisms include relieving inflammation (48, 51), decreasing the deposition of fibrin, down-regulating the expression of phospho-Akt (p-Akt), and up-regulating the expression of myelin basic protein (MBP) (51).

### The Application of Batroxobin in Peripheral Vascular Diseases

Batroxobin treatment alone or in combination with other anticoagulant drugs could promote complete recanalization and prevent the incidence of postoperative deep venous thrombosis (DVT) without adverse events such as pulmonary embolism (PE) and hemorrhage (5, 25, 26, 64). Also, injection of Batroxobin with long-term micropump may get a better efficacy for DVT (27). Batroxobin in combination with aspirin also prevented restenosis after arterial angioplasty which may be mediated by decreased regional inflammation (4, 28–30, 32). In patients with atrial fibrillation (AF), Batroxobin improved blood rheology, decreased blood cell aggregation, and prevented left atrial thrombus formation (34).

In peripheral vascular-related animal models, Batroxobin improved hemostasis (56, 57, 67), and prevented thrombosis (54, 58), accelerating tissue repair and vascular regeneration and stabilizing the atherosclerotic plaque (59, 62). The effect of Batroxobin on fibrinogen metabolism played an important role in ameliorating the formation of disseminated intravascular coagulation (DIC) (60, 61). As an adjunct, Batroxobin enhanced the thrombolytic effects of native tissue-type plasminogen activator (nt-PA) (55). The role of Batroxobin in inhibiting human vascular smooth muscle cell (SMC) migration may also play a clinical value in the future (63).

### Cerebral Venous Sinus Thrombosis May Benefit More From Batroxobin

Timely diagnosis and treatment are essential for faster and more complete recanalization and better outcomes in patients with cerebral venous sinus thrombosis (68–70). However, the primary treatment of CVST is long-term oral anticoagulation. For acute and severe CVST, endovascular therapy is always used first (71). Whereas, venous recanalization is time consuming and there remains a risk of hemorrhagic transformation after anticoagulation. Further complications of endovascular interventions make these interventions a dilemma for most physicians. Therefore, exploration of optimized treatment strategies in CVST is necessary.

Hyperfibrinogenemia, decreased blood flow velocity, and increased viscosity of hyperfibrinogenemia are the three major factors that promote venous thrombosis (72). Batroxobin is a serine protease extracted from the venom of the snake *Bothrops atrox moojeni*, and it exerts defibrinogenating effects (13). Batroxobin reduces the concentration of fibrinogen in blood by degrading fibrinogen to fibrin degradation products (FDPs) and D-dimer (12, 13). The defibrinogenating effect of batroxobin improves microcirculation by reducing vascular resistance and increasing blood flow velocity (30). Batroxobin can also mobilize endothelial cells to release endogenous t-PA, which indirectly promotes thrombolysis (12, 13). Therefore, Batroxobin can play both preventative and therapeutic roles in pat without increasing the risk of bleeding events in patients with a high risk of CVST.

Despite the controversial effect of Batroxobin on coagulation status, the significant reduction of the amount of bleeding and the effect on hemostasis by Batroxobin was well studied. Batroxobin combined with anticoagulation can significantly promote the recanalization of CVST and cortical venous thrombosis (CCVT) without increasing the risk of bleeding (10, 11). Venous stasis and the embolism from the venous sinus, especially the superior sagittal sinus, were the main risks CCVT in CVT patients (73–75). CCVT is often secondary to venous infarct and hemorrhagic transformation. A previous study reported that Batroxobin reduced the death/apoptosis of damaged neurons, the size of the ischemic infarct, and the risk of bleeding conversion (36). Therefore, CCVT patients are likely to benefit from Batroxobin treatment. CVST or venous infarct-induced cerebral edema resulted in a series of clinical symptoms of intracranial hypertension, which is often a predictor of poor prognosis (75, 76). Previous studies showed that CVST patients benefit from decompressive craniotomy (77). However, decompressive craniotomy might be better suited for severe cerebral edema caused by large venous infarcts. For CVST patients with mild intracranial hypertension caused by edema, Batroxobin may be a better choice since it reduces tissue edema and inhibits cytotoxic damage, as demonstrated in previous studies (14, 15, 42, 65).

CVST patients always showed good neurological and cognitive long-term outcomes (78). However, some patients also presented with significant neurological impairment or neuropsychological deficits due to the disruption of functional areas or conduction tracts when the cerebral cortex is infarcted because of CVST or thrombosis in the deep cerebral venous sinus (75, 79). Cognitive dysfunction is an important factor affecting patients' quality of life and aggravating family burden. Therefore, in the acute stage of CVST or venous infarcts, intervention measures are needed to protect nerve cells in the damaged area to avoid or mitigate cognitive impairment as much as possible. Batroxobin improves free radical scavenging leading to neuroprotective function. A previous study reported that Batroxobin was effective in improving vascular cognitive impairment (VCI) caused by ischemic cerebrovascular disease after long-term treatment (24). Future studies are needed to investigate whether the cognitive dysfunction associated with CVST can benefit from the use of Batroxobin.

In summary, Batroxobin had broad clinical applications in both arterial and venous thrombosis, including promotion of thrombolysis, prevention of thrombotic formation, reduction of edema in infarcted areas, improvement of vascular cognitive dysfunction, and neuroprotection. The potential mechanisms include promotion of depolymerization of fibrinogen polymers, increase in the capacity of free radical scavenging, reduction of inflammation, and regulation of endogenous plasminogen activator expression. Batroxobin can also be therapeutic in CVST and their secondary diseases. However, the application of Batroxobin was still limited to clinical studies with small sample size. Future multi-centered studies with randomized design and larger sample size would provide more evidence on the potential effect of Batroxobin in cerebral vascular diseases.

## Conclusion

Batroxobin could treat both arterial and venous ischemic diseases by promoting depolymerization of fibrinogen polymers, regulating the expression of related molecules, reducing oxidative stress, and reducing the inflammation response. However, current evidence of the beneficial effect of Batroxobin in cerebral vascular diseases was mostly from clinical and experimental studies with small sample size and high heterogeneity. Multi-centered clinical trials with randomized design and larger sample size would be needed in the future.

## Author Contributions

DL and SYS: manuscript drafting and revision, study concept and design, collection, assembly, and interpretation of the data. BLJ: collection, assembly, and interpretation of the data. RM, YHL, and SYS: manuscript drafting and revision, study concept and design, deeply edited the revised version and contributed critical revision, and final approval of the manuscript.

## Funding

This work was supported by the National Key R&D Program of China under Grant (2017YFC1308400), the National Natural Science Foundation under Grant (81371289), and the Beijing Natural Science Foundation (7212047).

## Conflict of Interest

The authors declare that the research was conducted in the absence of any commercial or financial relationships that could be construed as a potential conflict of interest. The reviewer PW declared a shared affiliation, with no collaboration, with the authors to the handling editor at the time of the review.

## Publisher's Note

All claims expressed in this article are solely those of the authors and do not necessarily represent those of their affiliated organizations, or those of the publisher, the editors and the reviewers. Any product that may be evaluated in this article, or claim that may be made by its manufacturer, is not guaranteed or endorsed by the publisher.

## Supplementary Material

Supplementary Table 1 PRISMA 2020 checklist.
